# On the relationship between oil market and European stock returns

**DOI:** 10.1007/s11356-023-31049-8

**Published:** 2023-11-20

**Authors:** Cosimo Magazzino, Muhammad Shahbaz, Massimiliano Adamo

**Affiliations:** 1 Department of Political Science, Roma Tre University, Rome, Italy; 2 Department of International Trade and Finance, School of Management and Economics, Beijing Institute of Technology, Beijing, China; 3 Center for Sustainable Energy and Economic Development, Gulf University for Science and Technology, Mubarak Al-Abdullah, Kuwait; 4 Sapienza-University of Rome, Rome, Italy

**Keywords:** Oil market, European stock market returns, Time-varying causality, Time series, Europe, E44, G12, Q43

## Abstract

This paper investigates the dynamic relationship between the oil market and European stock market returns using monthly data from May 2007 to April 2022 for 27 European Union member countries. A novel approach is adopted by using the time-varying Granger causality test and the structural vector auto-regression model to examine the causal links. Empirical results reveal strong evidence of time-varying causation between the variables, considering the oil market from both the supply-side and demand-side perspectives. In light of these findings, numerous policy considerations emerge, including refining risk management strategies for investors, reformulating economic and energy policies, the potential impact on monetary policy decisions, the need for ad hoc market regulations, facilitating investor education initiatives, promoting international cooperation, and advancing the transition to sustainable energy sources.

## Introduction

The relationship between oil prices and stock market returns has been a subject of interest among researchers and practitioners in recent years, given that oil is a crucial natural resource that drives economic growth and development (Sardar and Sharma 2022).

Recent years have been marked by significant events in the oil market, which have created economic uncertainty and instability. One of the most significant events was the collapse in oil prices in 2020, caused by a combination of over-supply and weak demand due to the corona virus disease 2019 (COVID-19) pandemic crisis (Fattouh and Imsirovic 2020). This led to a sharp decline in oil prices, with Brent crude oil falling to below $20 per barrel in April 2020.

The COVID-19 pandemic exerted an unprecedented impact on the oil market, with global demand plummeting as lockdowns were imposed worldwide. The price of oil experienced a sharp fall, and at one point, the West Texas Intermediate (WTI) crude oil price even turned negative, which meant that producers were paying buyers to take the oil off their hands. This represented a clear contrast to the pre-pandemic market equilibrium, where the oil prices were over $100 per barrel. Thus, the pandemic-induced downturn, along with the over-supply of oil and a sort of “price war” between Russia and Saudi Arabia, contributed to the significant decline in oil prices (Ma et al. 2021).

Since then, the oil market has seen a gradual recovery, with prices currently hovering around $80 per barrel. However, uncertainty remained in the market, with experts warning about the possibility of a next price crash. The rise in oil prices has also raised concerns about inflation and its impact on the global economy, forcing monetary authorities all over the world to counteract. This market has also been affected by political events, such as tensions between the United States (US) and Iran, which have led to concerns about supply disruptions. In addition, the decision by the Organization of the Petroleum Exporting Countries (OPEC) and its allies, including Russia, to cut production in order to support prices represented a significant factor in the oil market in recent years (Känzig 2021).

Furthermore, there has been a growing interest in renewable energy sources, which has led to a shift away from fossil fuels, including oil. This raised several warnings on the long-term future of the oil industry, as countries and companies increasingly invest in renewable energy sources (Magazzino et al. 2022).

Given the significant role of the oil market in driving the global economy and its recent volatility, understanding its effects is crucial for policymakers, investors, and suppliers. In fact, the fluctuations in oil prices might have far-reaching consequences, affecting various sectors, including transportation, manufacturing, and energy. Therefore, studying the oil market and its repercussions on the economy can help in developing strategies to mitigate its impact and facilitate better decision-making. This understanding goes beyond mere information; it is a cornerstone for making well-informed decisions and developing approaches to navigate and alleviate the wide ramifications caused by oil market fluctuations. These effects ripple through a number of different sectors, including transportation, manufacturing, and energy, underscoring the critical nature of this examination.

Several studies have explored the relationship between oil prices and stock market returns using different econometric techniques. Nevertheless, the European case remains substantially unexplored. The Euro Stoxx 50 Index is a stock market index that includes 50 large, blue-chip companies from 12 Eurozone countries. It is one of the most widely followed stock indices in Europe, providing a good representation of the overall performance of the Eurozone stock market.

Since the Eurozone is a major economic region and a significant consumer of oil, the relationship between the Euro Stoxx 50 Index and oil prices is of great interest to researchers and policymakers. Understanding the dynamic relationship between these two variables can provide insights into the broader economy and help guide policy decisions related to energy independence, investment, and economic growth, also in the context of sustainable development and energy transition.

Fluctuations in oil prices can, therefore, exert a significant impact on the profitability and stock prices of companies that operate in these sectors, which are represented in the Euro Stoxx 50 Index. By analyzing the relationship between the Euro Stoxx 50 Index and the oil market, researchers can gain a better understanding of the broader economic implications of oil market movements and their impacts.

This study sheds new light on the relationship between crude oil prices and production, along with European stock market returns. Using a structural vector auto-regression (SVAR) model and time-varying Granger causality (TVGC) tests on a recent dataset spanning from May 2007 to April 2022, the analysis goes beyond the standard applied analyses. In fact, the causality from oil supply to the stock market has never been studied in Europe considering the whole continent.

Furthermore, the study presents a novel exploration of the correlation among crude oil prices, production, and European stock market returns. Notably, our analysis overcomes conventional approaches examining the causality between oil supply and the stock market throughout the entire European continent, an aspect previously unexplored. In particular, the analysis investigates the intricate cause-and-effect connections between oil supply and stock market behavior for 27 EU countries, an aspect roughly neglected in previous research. To this end, advanced empirical methodologies — such as the TVGC test — are employed. This fusion of cutting-edge techniques with a comprehensive dataset that consolidates information from all European nations allows us to unearth previously hidden insights and illuminate the complex dynamics underlying this multifaceted relationship.

In order to conduct a thorough evaluation of the relationship under investigation, we included in the empirical analysis multiple variables able to capture different aspects of the phenomenon. Specifically, we included the percentage change of non-European and European oil production, which is measured in thousand barrels per day. This enables us to assess the impact of oil supply. In addition, we use the real economic activity index, which was initially introduced by Kilian and Park (2009), as a reliable measure of global demand within the industrial commodity market. By incorporating this index, we aim to capture the broader economic factors influencing the relationship between crude oil prices and production. To gauge oil-specific demand, we also selected Brent oil prices, to examine the direct influence of price fluctuations. Additionally, we consider the returns of the Euro Stoxx 50 Index, which serves as a robust indicator of the stock market performances. This variable provides valuable insights into the impact on the financial realm.

Thus, by incorporating these variables into the analysis, we aim to provide a comprehensive understanding of the interconnections among crude oil prices, production, and European stock market returns.

In this study, innovative stationarity and unit root tests are employed, together with the TVGC tests, to examine the time series properties of the selected variables. The findings of the analysis make a significant contribution to the existing literature in several ways: first, by exploring the relationship between the European stock market, without any separation between national indexes and the oil prices; second, by distinguishing between the demand and supply sides of the oil market; third, by creating a variable that summarizes the European oil production variations including all European Union (EU) member countries and the United Kingdom (UK) until Brexit; and fourth, by highlighting the inextricable link between the global supply of oil and European markets, a topic that has not been explored for the European case. Overall, these findings provide a novel and valuable insight into the interactions between these relevant economic factors.

The rest of the study is as follows: “Overview of the literature” section provides an overview of the relevant literature. “Methodology, model, and data” section describes the methodology used, the estimated model, and the dataset. “Empirical results and discussion” section presents and discusses the empirical findings, showing recent trends in oil prices and production movements and their impact on the European stock market. “Conclusions and policy implications” section concludes by suggesting relevant policy implications, including the need for policymakers to consider the impact of oil prices and production fluctuations on the global economy and to develop strategies to mitigate their effects.

## Overview of the literature

The amount of research related to the relationship between oil prices and stock returns is vast. Previous studies followed different methodologies and implemented alternative empirical strategies, resulting in significant variations in their findings depending on the data and study period.

To provide a comprehensive understanding of the topic, this section presents a concise overview of the most relevant contributions in the field, starting with the research conducted in the US. Kilian and Park (2009) developed a VAR model using global oil production, a real economic activity index, oil prices considered as the costs paid by US refiners, and the CRSP equally weighted portfolio for US stock returns. The time span is 1973–2006. Empirical findings show that the reaction of the US real stock returns to a shock to the oil prices can differ heavily depending on whether the change in this price is caused by the demand or the supply in the relevant market. Both types of shocks accounted for almost 22% of the long-run variation in the US real stock returns.

Recently, Känzig (2021) analyzed how changes in oil supply expectations affect the oil price and the macroeconomy using high-frequency data from 2003 to 2017, for the OPEC member states. Sardar and Sharma (2022) investigated the nonlinear relationship between oil prices and the US stock returns around the zero lower bound (ZLB) between October 1987 and March 2020. Findings suggest that oil price shocks lead to higher stock returns during the ZLB. Shahzad et al. (2022) explored the evolution of volatility of the Bloomberg commodity index and WTI crude oil prices at different time scales using wavelet analysis (WA), showing that these variables are correlated in the medium to long term but not in the short term. Using a similar methodology, Mutascu et al. (2022) inspected the co-movements of gasoline and diesel prices in three European countries (i.e., Germany, France, and Italy) with different fuel tax systems, through a weekly dataset over the period January 2005–June 2021, providing empirical evidence of co-movements between gasoline and diesel at all frequencies.

Kang et al. (2016), using an SVAR model with two different data periods (the first from 1973 to 2006 and the second from 1972 to 2014), demonstrated the significance of distinguishing between shocks to the US and non-US oil supply when analyzing the effects of structural shocks on the US real stock increases. They concluded that one supply-related shock can affect the US stock returns with a decomposition of the global supply into US and non-US. Moreover, they found that there is a positive correlation between oil output shocks and US real stock returns. This is an important outcome, which emphasizes how crucial it is to distinguish between the effects of domestic and international oil production on current stock gains in recent years. Contrarily, Kilian and Park (2009) found that when supply shocks from domestic and international oil production are recognized, oil demand and supply shocks are of equivalent importance in explaining the US real stock returns.

Foroni et al. (2017) used structural analysis with data from 1973 to 2015 to show how the relationship between oil prices and US equity returns varies over time. According to a reduced-form analysis, a link between equities returns and the price of oil emerges after the financial crisis. In addition, the study revealed that fluctuations in oil-specific demand had favorable effects on the US stock market, compared to oil supply shocks, which had little impact on market returns since 2008. Additionally, the study showed how the SVAR parameters change over time.

Regarding studies on European countries, the research focus shifts from aggregate stock market returns to sector-specific ones. Arouri (2011) investigated the responses of European sector stock markets to changes in oil prices through a linear and asymmetric model. Strong and significant links between oil prices and most European stock market returns emerge, depending on the sector. These findings emphasize the importance of considering sector-specific effects when analyzing the relationship between oil prices and European stock market returns.

Sadorsky (1999), using a VAR model with the Cholesky factorization, modelled the oil prices volatility with a generalized auto-regressive conditional heteroskedasticity (GARCH) model. It emerges that oil prices have a significant negative impact on European stock markets for several European countries looking at the impulse-response functions (IRFs). Cunado and De Gracia (2014) separated the stock market returns by countries instead of sectors and used global oil production as an aggregate. The results obtained, modelling variables from 1973 to 2011 with VAR and vector error correction model (VECM), highlight a negative impact of oil prices on stock returns.

Park and Ratti (2008) analyzed a dataset from 1986 to 2008 looking at the volatility of oil prices by applying Baba-Engle-Kraft-Koner GARCH and dynamic conditional correlation (DCC)-GARCH models. The median result from forecast error variance decomposition (FEVD) is that oil prices account for almost 6% of volatility in stock returns for different European countries. Kang et al. (2015) estimated a VAR model including a measure of uncertainty extrapolating the Google searches and the consumer price index. The sample covered the 1985–2011 period, and it is found that oil market-specific demand shock accounts for 30% of economic policy uncertainty after 24 months and 58% in the long term. Kang et al. (2015), with a time-varying VAR model, studied the effects of oil shocks on the US stock market return with data from 1973 to 2013. The contribution of oil-specific demand shocks rose from 5% in the 1970s to about 15% in 2007, then a decline was registered; while the oil supply shocks’ effects had a decreasing trend, from 17% in the 1970s to 5% in 2012. Mokni (2020) explored the time-varying relationship between the oil and stock markets of oil-importing and oil-exporting countries. Using an SVAR model and a time-varying parameter regression with monthly data from 1999 to 2018, evidence of time-varying effects of stock market returns to different oil shocks (specifically demand shocks) is found. In particular, oil supply shocks are limited and negative, while oil-specific demand shocks have positive effects on the oil-exporting stock returns and negative effects on the oil-importing countries.

With a different perspective, Mohanty et al. (2010) tried to estimate the effects of oil shocks on the stock returns of Central and Eastern European firms related to the oil and gas sector. The sample period covered the years 1998–2010. The empirical findings evidence a link between the two variables, but only in sub-periods. Bein and Aga (2016) developed an analysis of national stock market returns and the effects caused by the oil market, using stock market returns from Nordic countries (i.e., Denmark, Finland, Sweden, and Iceland) for the 1995–2015 period. The empirical strategy used a DCC-GARCH model with a stock market modelled through GARCH and a Markov switching auto-regressive model for oil prices. A correlation between all European stock market indices and Brent and WTI oil prices is found.

Arouri et al. (2011) aimed to capture the relationship between oil prices and aggregate and sectorial stock market returns in Europe using a VECM model with an asymmetric cointegration approach. The dataset covered the 1998–2008 years, with evidence of a relationship both for aggregate and sectorial stock indices. In Arouri and Jawadi (2010), a VAR model investigating the oil price shocks on 12 European sectorial stock markets is presented. Based on weekly data from 1998 to 2008, the short-term analysis found a strong significant nexus between oil price changes and the stock market for most European sectors. Degiannakis et al. (2013) established a time-varying correlation between oil prices and European industrial sector indices using a multivariate ARCH model with a time-varying parameter on a sample from 1992 to 2010. Degiannakis et al. (2014) highlighted that the shocks to the aggregate demand trigger negative effects on oil price volatility, while the specific oil demand shock does not affect the Euro Stoxx 50 Index volatility. The study employed an SVAR model with a constructed sectorial index, global oil production changes, global economic activity, and Brent prices, using daily data from 1999 to 2010. Degiannakis et al. (2018) proposed a time-varying VAR model using oil price changes, stock returns (aggregate and sectorial), global oil production, industrial production, interest rates, and unemployment rate. The results for the 1990–2015 period show a time-varying relationship. Similarly, including some macroeconomic variables in the analysis, Park and Ratti (2008) estimated a VAR model with monthly data from 1986 to 2005 using interest rates, real oil price changes, industrial production, and real stock returns for 13 EU countries. It is shown that oil prices play a very important role in determining the stock market of oil-importing countries. On the other hand, in oil-exporting countries, oil price shocks have a much smoother impact on the stock market rather than an interest rate shock. Finally, monetary policy does not seem to respond to oil price shocks.

A different strand of literature focused on causality analysis. Katsampoxakis et al. (2022) investigated the link between Brent oil prices and importer and exporter European national stock indices (RTS, OBX, CAC40, DAX), analyzing only the COVID-19 pandemic period (2019–2020). Applying a VAR model and Granger causality tests, changes in the relationship from pre- and post-pandemic period are highlighted. In the steady phase with low volatility — such as pre-pandemic and post-vaccination periods — no causality is established, while when the market volatility is higher, causality appears. Before the COVID-19 pandemic, only OBX index from Norway (an oil net-exporting country) exhibited a symmetric causality flow with oil prices. During the vaccination period, DAX and CAC40 caused oil prices. Finally, for the post-vaccination period, OBX, RTS, and CAC40 are found to cause oil prices. Agarwalla et al. (2021) used a VECM to model the Indian stock index, with data from September 2005 to March 2015. Granger causality tests show evidence of causality from oil prices to stock returns. Tawfeeq et al. (2019) investigated the causality flow between stock returns and oil prices in Middle East countries modelling data from 2001 to 2015 with VAR and VECM. The results showed short-term causality from oil prices to market capitalization in two countries during the specified time period and in five countries during the pre-shale period, and six countries during the post-shale period. Furthermore, the IRFs provided evidence of the correlation between oil prices and stock market value in most of the Middle Eastern markets studied. Abubakirova (2021) studying a dataset from January 2010 to December 2019 analyzed the symmetrical and asymmetrical causality between stock and Brent prices for Brazil, Russia, India, China, and South Africa countries. For Brazil, Russia, and South Africa, a symmetrical relationship is found from oil prices to stock prices. An asymmetrical causation is established for Russia, with a positive causal flow running from stock prices to oil prices, while China and India exhibit a negative bi-directional causal link (feedback mechanism). Daradkah et al. (2021) investigated the connection between stock market returns and oil prices in three MENA oil-importing countries (Egypt, Morocco, and Jordan) from 2005 to 2018. The study used VAR estimates, Granger causality tests, and IRFs to examine the relationship between the series. The results show a causal relationship from oil prices to stock market returns in all three countries, but with different time lags and response patterns based on IRFs outcomes.

Recently, Atif et al. (2022), using a panel VAR analysis, inspected the relationship between oil prices and stock returns for big economies, including both oil-importing and oil-exporting countries, with data between January 2019 and December 2020. Empirical results evidence how stock indexes are caused by oil prices, as well as a symmetric relationship between the variables of interest.

Serrano and Angosto-Fernández (2022) conducted an analysis on how the Russian-Ukrainian war impacted global equity returns in the short term. Using event study and regression analysis on 77 global capital markets to determine the short-term impact of an event, together with cross-sectional methods to determine its size and variability, the study finds that the impact of the event is significant on and around day zero. It also highlights factors that affected investors, such as being in Soviet orbit and the North Atlantic Treaty Organization simultaneously, high gas consumption, and importing gas from Russia.

Raifu (2023) explored the complex interaction between oil yields and stock returns in Norway using LA-VAR and TVGC for the 2011–2021 period, revealing that the frequency of data collection plays a key role in determining the direction of causality between these two financial parameters. In particular, a two-way causality is discovered between oil returns and stock returns, indicating a dynamic interaction. Moreover, the existence of time-varying causality is also established. This dynamic aspect of the association underscores that the causal connection between oil and stock returns is far from static, highlighting an evolution over time (Table 1).

**Table 1:** Summary of existing literature on oil market shock’s effects on stock market returns

Author(s)	Study period	Empirical strategy
Abubakirova (2021)	2010–2019	VAR, GC
Agarwalla et al. (2021)	2005–2020	VECM, GC
Arouri (2011)	1998–2010	LSM
Arouri and Jawadi (2010)	1998–2008	VAR
Arouri and Rault (2012)	1998–2010	MM
Arouri et al. (2011)	1998–2008	VECM
Atif et al. (2022)	2019–2020	PVAR, GC
Bein and Aga (2016)	1995–2015	DCC-GARCH, Markov switching AR
Cunado and De Gracia (2014)	1973–2011	VAR, VECM
Daradkah et al. (2021)	2005–2018	VAR, GC
Degiannakis et al. (2013)	1992–2010	M-ARCH
Degiannakis et al. (2014)	1999–2010	SVAR
Degiannakis et al. (2018)	1990–2015	TV-VAR
Serrano and Angosto-Fernández (2022)		Event study
Foroni et al. (2017)	1973–2015	TV-VAR
Kang et al. (2015)	1985–2011	VAR
Kang et al. (2015)	1973–2013	TV-VAR
Kang et al. (2016)	1972–2014	SVAR
Katsampoxakis et al. (2022)	2019–2020	VAR, GC
Kilian and Park (2009)	1973–2006	SVAR
Känzig (2021)	2003–2017	SVAR
Mohanty et al. (2010)	1998–2010	Oil price risk sensitivity analysis
Mokni (2020)	1999–2018	SVAR, TVR
Mutascu et al. (2022)	2005–2021	WA
Park and Ratti (2008)	1986–2005	VAR
Park and Ratti (2008)	1986–2008	BEKK-GARCH, DCC-GARCH
Raifu (2023)	2011–2021	LA-VAR, TVGC
Sadorsky (1999)	1947:1–1996:4	
Sardar and Sharma (2022)	1987–2020	SDLP
Shahzad et al. (2022	October 24, 2005–May 4, 2016	WA
Tawfeeq et al. (2019)	2001–2015	VAR, VECM, GC

*BEKK-GARCH* Baba-Engle-Kraft-Koner generalized auto-regressive conditional heteroscedasticity, *CVAR* Cholesky vector auto-regression, *DCC-GARCH* dynamic conditional correlation generalized auto-regressive conditional heteroscedasticity, *GC* Granger causality, *LSM* linear asymmetric model, *M-ARCH* multivariate auto-regressive conditional heteroscedasticity, *MM* multifactor model, *MS-AR* Markov switching auto-regressive model, *PVAR* panel vector auto-regression, *SDLP* state dependent local projection, *LA-VAR* lag augmented vector auto-regression, *SVAR* structural vector auto-regression, *TVR* time-varying regression, *TV-VAR* time-varying vector auto-regression, *VECM* vector error correction, *WA* wavelet analysis

Source: author’s elaborations

## Methodology, model, and data

This section illustrates the empirical strategy followed in the study. To test for stationarity, four tests were conducted: the augmented Dickey-Fuller (ADF) test, the Dickey-Fuller generalized least squares (DF-GLS) test, the Leybourne test, and the Elliott, Rothenberg, and Stock (ERS) unit root test.

The Leybourne test has been used to identify unit roots under various conditions, making it a valuable analytical tool. It is noteworthy that, to improve the sensitivity of the unit root test, we applied a local-unit detrending technique, as proposed by Elliot et al. (Elliott et al. 1996). In addition, the ERS test introduced the P-test, a practical and optimal evaluation method that takes into account the serial correlation of error terms. The DF-GLS test, similar to the ADF test, was applied to detrended data without incorporating an intercept. This approach provides a complementary perspective to the overall examination of data stationarity.

Then, an SVAR model has been implemented. SVAR model differs from VAR since it takes into account contemporaneous relationships between variables. This is beneficial in analyzing the IRFs and FEVDs, as it reveals the immediate effect of a shock on different variables.

Blanchard and Quah (1989) developed a method that avoids direct restrictions on the structural matrices. Instead, it looks at the structural innovations and imposes no restrictions on their cumulative relationships.

After estimation, the first step in post-analysis is to examine the stability of the eigenvalues. The stability of the estimated VAR model is assessed by checking if the modulus of each eigenvalue is less than one, as suggested by Lutkepohl (2005) and Hamilton (1994). The analysis includes a Wald test to verify if all endogenous variables at a given lag are zero, and a Lagrange multiplier test to detect autocorrelation in the residuals. The normality of disturbances is evaluated by the Jarque–Bera test statistic. Furthermore, IRFs and FEVDs are used to analyze the effects of shocks on endogenous variables.

To capture time variation in Granger causal orderings and to isolate the timing of changes, recursive estimation methods are further employed. Three algorithms generate a sequence of test statistics: forward expanding (FE) window, rolling (RO) window, and recursive evolving (RE) window. The analysis considers a sample of *T* + 1 observations, a number *r* such that 0 < *r* < 1, and the term [*Tr*] to indicate the integer component of the product.

In the FE algorithm (Thoma 1994), a standard forward recursion is performed by computing the Wald test statistic first for a minimum window length, *τ*_0_ = [*Tr*_0_], and then expanding the sample size sequentially by one observation until the final test statistic is computed using the entire sample. At the end of the FE algorithm, a sequence of Wald test statistics, *Tr*_1_, *r* with *r*_1_ = 0 and *r* ∈ [*r*_0_, 1], is obtained.

The RO algorithm (Swanson 1998; Arouri and Rault 2012) advances one observation at a time through a window of size [*Tw*] while computing a Wald test statistic for each window. The output from the RO algorithm is a sequence of test statistics *Tr*_1_, *r* with *r*_1_ = *r − w* and *r* ∈ [*r*_0_, 1], where each test statistic is computed from a sample of the same size, [*Tw*] with 0 < *w* < 1.

In the RE algorithm, for a given observation of interest, a test statistic is computed for every possible subsample of size *r*_0_ or larger, with the observation of interest providing the common endpoint of all subsamples. This procedure is repeated for every observation in the sample, subject only to the minimum window size. As a result, every observation in the sample after the first one has a set of Wald test statistics attached. The inference is based on a series of these statistics’ supremum norms, according to Phillips et al. (2015).

The statistic’s supremum norms refer to a sequence of maximum values for the Wald test statistics generated by the RE algorithm, where the algorithm computes a test statistic for every possible subsample of a given size or larger. Thus, the RE algorithm produces a sequence of test statistics *T*_*r*1*, r*_ with *r*_1_ ∈ [0, *r* − *r*_0_] and *r* ∈ [*r*_0_, 1], which are the supremum norms of the Wald statistics for every single observation. The RE algorithm encompasses both the FE and RO recursions as special cases.

For each observation, a sequence of test statistics is defined and can be arranged in an upper triangular square matrix with column and row dimensions equal to the largest number of usable observations. The FE Wald statistic is the leading entry in each column, the RO Wald statistic is located on the main diagonal, and the RE statistics make up the bulk of each column. The obtained information can be applied to the entire sample or used to focus on the timing of these time-varying events through period analysis. If the null hypothesis of interest is whether a particular variable does not Granger cause another variable at any time during the sample, with the alternative that there is Granger causality at some time, a single test statistic is required. The maximal FE statistic is the largest element of the first row of the upper triangular matrix of the test.

The dataset used in this study covers the period from May 2007 to April 2022, with monthly observations. The oil supply is divided into domestic and foreign; European and non-European production variables were obtained from the US Energy Information Administration, measured in thousand barrels per day. European production was constructed by aggregating the single oil production of the 27 EU member countries plus the UK, which was included until January 31, 2020, the date of the Brexit process conclusion. Non-European production is the world’s production minus European production. Both oil production variables have been taken in percentage change.

The aggregate demand component was calculated using the index created by Kilian and Park (2009), which was constructed to consider all the economic changes building an index on cargo shipment. This is a monthly measure of global real economic activity designed to capture cross-sectional changes in global demand for industrial commodities. The index is derived considering an equal-weighted index of percentage growth rates obtained from a panel of single-trip dry bulk ocean freight rates, measured in dollars per ton.

The rationale for using Kilian’s index is that an increasing spike in dry bulk ocean freight rates will be indicative of an increase in demand for shipping services resulting from the increased global real activity. A major advantage of this monthly index based on dry bulk sea freight rates is that it automatically incorporates the effects of increased real activity in newly emerging economies such as China or India, for which monthly industrial production data are not available. In contrast, more conventional measures of monthly global real activity such as the OECD industrial production index exclude real activity in China and India. Kilian’s index can be derived from the Federal Reserve Bank of Dallas (in first differences).

The Brent crude oil monthly closing price at log-levels was used as the price of oil in order to have a clue of the specific oil demand; it is taken from St. Louis FED. Lastly, the European stock returns were taken by calculating the returns of the Euro Stoxx 50 Index, taken from Yahoo Finance. Table 2 summarizes the main features of the dataset.

**Table 2:** Variables’ description

Variable	Abbreviations	Measurement units	Data source
Non-European oil production	NOEUOP	Percentage change of thousand barrels extraction per day	EIA
European oil production	EUOP	Percentage change of thousand barrels extraction per day	EIA
Real economic activity index	REAK	First difference of dollar-denominated global bulk dry cargo shipping rates	Dallas FED
Brent oil monthly closing price	Brent	Natural log of the monthly closing price in US dollars	St. Louis FED
Euro Stoxx 50 Index monthly returns	EURet	US dollars	Yahoo Finance

Sources: authors’ elaborations

1. https://www.eia.gov/dnav/pet/pet_crd_crpdn_adc_mbbl_m.htm

2. https://www.eia.gov/dnav/pet/pet_crd_crpdn_adc_mbbl_m.htm

3. https://www.dallasfed.org/research/igrea

4. https://fred.stlouisfed.org/series/MCOILBRENTEU

5. https://finance.yahoo.com/quote/%5ESTOXX50E/?guccounter=1&guce_referrer=aHR0cHM6Ly93d3cuZ29vZ2xlLmNvbS8&guce_referrer_sig=AQAAAKT6sddy3DfnlgIIWlqNjbmGrwMqyEl3LREbxhzbSJsTENGDXFw7tn_mVou1gfBhVIRZS_PY9lQFImOeVG9_QNZy5iuSWOi37C7gZRy1xwEzSILK0T_ThmwDxjlwU_KbNG91wmrSwCUTrSA_Qknuewda4bhcoht2iF7eSdSnTXcW

## Empirical results and discussion

Table 3 displays the descriptive statistics of the variables. The results are generally consistent with expectations, except for the extreme values observed for *NOEUOP* (European oil production) in terms of skewness (− 5.11).

**Table 3:** Descriptive statistics

Variable	Mean	Median	Std. dev	Skewness	Kurtosis	Range	IQR	CV
NOEUOP	0.1047	0.2194	1.2014	− 5.1112	51.2001	15.1813	0.8919	11.4764
EUOP	− 0.3475	− 0.3151	7.2344	− 2.8414	27.0016	80.6336	6.4576	− 20.8162
REAK	− 5.0246	− 16.6350	73.9719	0.7685	3.2461	351.2483	92.9608	− 14.7220
Brent	4.2791	4.2900	0.3662	− 0.4908	2.9788	1.9770	0.6117	0.0856
EURet	− 0.0008	0.0058	0.0527	− 0.4799	4.0413	0.3439	0.0672	− 65.7727

*Std. dev.* standard deviation, *IQR* inter-quartile range, *CV* coefficient of variation

Sources: authors’ calculations

Table 4 presents the stationarity analysis, which includes the results of ADF, DF-GLS, Leybourne, and ERS tests. The findings indicate the presence of a unit root for the real economic activity index (*REAK*), while the remaining variables (NOEUP, EUOP, Brent, EURet) are stationary, since the null hypothesis is soundly rejected at any conventional level of significance.

**Table 4:** Results for unit roots and stationarity tests

Variable	ADF	DF-GLS	Leybourne	ERS
NOEUOP	− 10.576*** (− 2.885)	− 10.363*** (− 2.043)	− 13.746*** (− 2.507)	− 13.707*** (− 2.082)
EUOP	− 10.606*** (− 2.885)	− 8.517*** (− 2.043)	− 15.933*** (− 3.252)	− 15.204*** (− 3.018)
REAK	− 3.029** (− 2.885)	− 1.184 (− 2.026)	− 1.849 (− 2.507)	− 1.184 (− 2.082)
Brent	− 2.527 (− 2.885)	− 2.494** (− 2.037)	− 2.512** (− 2.507)	− 2.494** (− 2.082)
EURet	− 10.266*** (− 2.885)	− 4.365*** (− 2.032)	− 12.207*** (− 2.507)	− 4.365*** (− 2.082)

*p < 0.10; **p < 0.05; ***p < 0.01. 5% Critical values in parentheses

Based on the stationarity test results, an SVAR model is constructed. To determine the appropriate lag-length, the likelihood ratio, final prediction error, Akaike information criterion, Hannan–Quinn information criterion, and Schwarz’s Bayesian information criterion are used. A model with two lags is chosen following the majority of statistics, but also to select the most parsimonious model.

Tables 5 and 6 present the results of the TVGC tests. Table 5 shows the test results with European market returns as the dependent variable. We found evidence of Granger causality from oil production (European and foreign) to market returns at a 95% confidence level. The causal flow exhibits an upward trend throughout the study period, peaking in the first few months of 2020, and then sharply declining, likely due to COVID-19 restrictions that hit production in different sectors and deeply affected the oil market. Although oil output experienced a decline, the drop-in causation with domestic production was less severe and rebounded more quickly. This finding is novel in the literature. One possible explanation could be due to the energy market sensitivity in the continent, as it heavily relies on imports and has few countries that develop efficient energy sources. Therefore, oil output plays a significant role in shaping market expectations.

**Table 5:** Time-varying LA-VAR Granger causality test (dependent variable: EURet)

Variable	Max_Wald_forward	Max_Wald_rolling	Max_Wald_recursive
NOEUOP	3.230 (13.957)	15.426** (13.527)	15.426** (13.957)
EUOP	10.118** (8.992)	7.721* (9.136)	10.861** (9.276)
REAK	6.536 (8.821)	8.515* (8.993)	8.515* (9.322)
Brent	4.282 (12.085)	9.334* (12.398)	9.496* (12.398)

*p < 0.10; **p < 0.05; ***p < 0.01. 95th percentile of test statistics in parentheses

**Table 6:** Time-varying LA-VAR Granger causality test (dependent variable: Brent)

Variable	Max_Wald_forward	Max_Wald_rolling	Max_Wald_recursive
NOEUOP	1.930 (17.316)	12.879 (17.148)	12.879 (18.842)
EUOP	3.903 (10.430)	4.855 (10.934)	7.613 (11.312)
REAK	7.580 (10.082)	14.675** (10.178)	16.497** (10.494)
EURet	12.429*** (8.980)	11.899** (9.324)	14.293*** (9.861)

*p < 0.10; **p < 0.05; ***p < 0.01. 95th percentile of test statistics in parentheses

Additionally, stock returns are also caused by real economic activity and Brent prices (at a 10% confidence level). The causal relationship between the real economic activity index and European stock market returns appears to be inevitable because of the nature of Kilian’s index. The purpose of this index is to provide a summary of global economic activity, which enables a causal relationship with the most traded European index. Previous studies (i.e., Kilian and Park 2009; Kang et al. 2016; Foroni et al. 2017) found a significant impact of real economic activity index shocks on market returns. This result was based on the analysis of the IRFs. Studies focused on the US market particularly highlighted this effect, despite the fact that causal relationships between the two were not explored previously. In addition, the causal flow between Brent prices and stock market returns supports previous findings regarding the impact of oil output on the stock market, highlighting the sensitivity of the European stock market to events affecting oil prices. This result was observed in multiple studies. Significantly, this causal relationship showed distinct patterns in the different periods. It was most prominent in the early 2020s, coinciding with the onset of the global COVID-19 pandemic and the subsequent implementation of blocking measures that disrupted economic activities on a global scale. During this phase, the causal link showed a remarkable intensification.

However, with the latter part of 2020 and the early part of 2021 characterized by the evolution of pandemic responses and the spread of vaccination, we observed a sensible attenuation of the strength of the causal link. These fluctuations in the causal relationship offer valuable insights into the nuanced dynamics governing the relationship between oil prices and European stock market returns. They underscore the intricate interplay between external events and financial market responses.

**Fig. 1:**
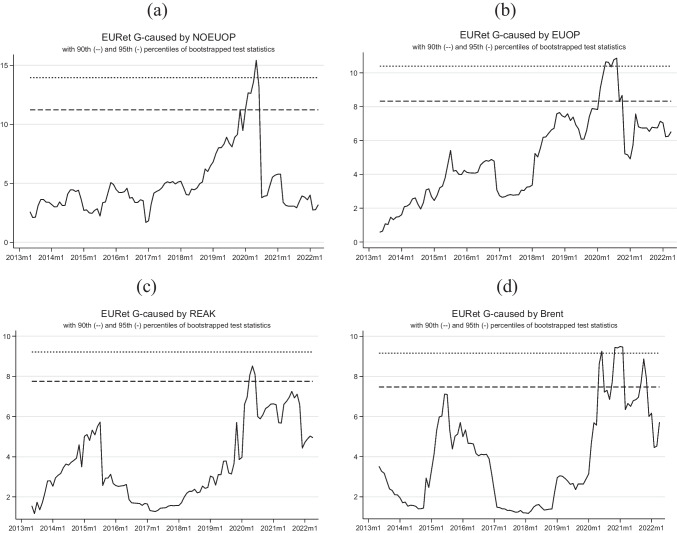
Recursive expanding Wald tests (dependent variable: EURet, 2007m6–2022m4). Notes: authors’ elaborations in STATA

Agarwalla et al. (2021) reported that, in the short term, the VEC Granger causality analysis showed a significant impact of international crude oil price movements on the Indian stock market. Daradkah et al. (2021) showed a causal relationship from oil prices to stock market returns in all three countries analyzed. It should be emphasized that these findings were specific to countries that import oil, a condition similar to the EU. Atif et al. (2022) showed a symmetrical relationship between oil prices and stock prices in Brazil, Russia, and South Africa, with causality running from oil prices to stock prices (Fig. 1).

Moving to Table 6, we conduct the TVGC tests using Brent prices as the dependent variable. In this case, more familiar outcomes emerge.

Granger causality test findings show no significant effect between oil production and Brent prices. The causality between foreign oil output and Brent prices was only marginally significant for a few months in 2019 and declined sharply in 2020. There was no significant causation found at a 90% confidence level for domestic production. Känzig (2021) stated that oil supply expectations can affect oil prices in the US. Expectations regarding oil supply can impact oil prices, reflecting market perceptions of future supply and demand imbalances. Indeed, if expectations of a shortage of supply emerge, for example, prices are likely to increase as traders and speculators anticipate higher demand and a corresponding price increase.

On the other hand, changes in oil production may not directly Granger cause oil prices because there may be a time lag between production changes and their effects on supply and demand. Granger causality framework is rooted in the idea that if a time series is causing another series to change, there should be a significant relationship between them, with the first variable occurring before the second. However, in the case of oil production and prices, it may take time for changes in production to affect supply and demand, and ultimately prices. Therefore, while changes in production may eventually affect oil prices, they may not have an immediate causal effect, at least in the sense of Granger.

**Fig. 2:**
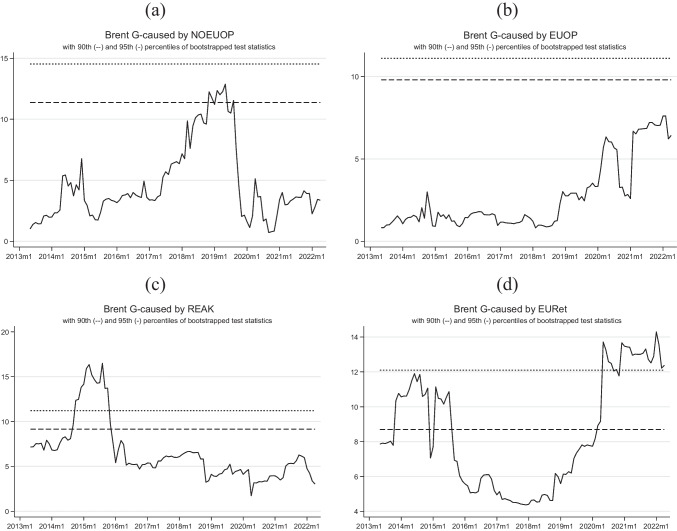
Recursive expanding Wald tests (dependent variable: Brent, 2007m6–2022m4). Notes: authors’ elaborations in STATA

*REAK* Granger causes Brent prices, which appears rather straightforward. In fact, this index aims to encapsulate trades encompassing raw materials, implying that movements in raw materials would also affect oil prices. The progression of this causality over the years is evident. Figure 2c shows a spike in 2014–2015 and a fall in 2016. Although this result has never been found in terms of causality, several studies have identified this relationship by analyzing the IRFs from a VAR or SVAR model. Kilian and Park (2009) emphasized this result, noting how a shock in the aggregate demand (represented by the REAK index) would have a significant impact on the oil-specific demand shock (represented by oil prices).

Then, we discover that *Brent* is caused by stock market returns at a 99% confidence level (for 2 out of 3 algorithms). This result is consistent with the literature, given the characteristics of the economies under examination. In 2013, there was a sharp increase, followed by a couple of years at a high level, and then a decrease in the last months of 2015. Between 2016 and 2018, no significant movements were observed.

It is worth noticing that we are dealing with a net importing region, which may explain why causality runs from market returns to oil prices. According to Atif et al. (2022), where both importing and exporting countries are inspected, a symmetric causality is observed. Katsampoxakis et al. (2022) highlighted that the market returns of oil-importing countries are found to cause oil prices with a unidirectional link. This was observed in the OBX index during the pre-COVID-19 period, in DAX and CAC40 during COVID-19 but before the vaccination campaign, and in CAC40, OBX, and RTS indexes after the pandemic. These results are in line with the findings of Abubakirova (2021), who discovered an asymmetric causality between stock prices and oil prices across multiple countries. They showed that the Russian national index returns positively affect oil prices, while a negative causality was observed between the national indices of India and China. These results provide evidence for the existence of a complex relationship between stock prices and oil prices in different countries, highlighting the need for further research to better understand the dynamics of this relationship. Agarwalla et al. (2021), and Tawfeeq et al. (2019), analyzing mainly exporter countries from the Asian and Middle Eastern regions, provided evidence of a causality nexus from oil prices to stock markets.

In the context of Europe, which is a net importer of oil, it is consistent for the heaviest and more significant causality to exist from the stock market to oil prices, despite the presence of symmetric causality between them. This is because changes in the price of oil have a comparatively lesser impact on the economy than changes in the stock market.

When the stock market rises, it indicates positive expectations about the economy, leading to an increase in the demand for oil and a subsequent increase in its price. Conversely, a fall in the stock market signals negative expectations, leading to a decrease in the demand for oil and a reduction in its price.

Thus, the stock market can be regarded as a leading indicator for the economy, including its demand for oil. In contrast, changes in oil prices may affect the economy, but not as directly or immediately as changes in the stock market.

In Table 7 in the Appendix, the correlation matrix of the variables is reported. We can see evidence of a negative correlation (with a *p*-value < 0.01) between *REAK* and *Brent*.

## Conclusions and policy implications

This paper presents an in-depth analysis of the relationship between oil prices and European stock returns. The empirical strategy implied an SVAR model and TVGC analyses, using monthly observations from 2007 to 2022 for both non-European and European oil production variations, the real economic activity index, Brent prices, and the Euro Stoxx 50 Index returns.

One of the key findings of this study is that there exists a causal link from oil prices to the European stock returns.

Furthermore, Brent prices seem to be caused by market returns, which aligns with previous literature. It is worth noting that oil-importing countries are generally not affected by market returns due to oil prices, but rather tend to cause changes in oil prices.

It is essential to consider that Europe is primarily an oil-importing region. This observation is even more pronounced after Brexit, as the UK was one of the main producers along with Norway and other Nordic countries. Therefore, most EU countries rely heavily on oil acquisition contracts, which makes energy independence an even more pressing concern. This study sheds light on the importance of finding alternative sources of energy to reduce dependence on imported oil, as it can have a significant impact on stock returns and overall economic activity.

The study’s findings carry significant policy implications, offering valuable perspectives on the intricate interconnection between the oil market and European stock market performance. These implications span a wide range of policy areas:

- Risk management for investors: this new knowledge equips investors with the means to make informed decisions, including portfolio diversification and risk mitigation strategies.
- Economic policy flexibility: policymakers may consider adapting economic policies to how oil market fluctuations affect the broader economy. Understanding the causal relationship between oil prices and stock markets can guide governments in creating measures to protect the entire economy from any negative effects of changes in oil prices.
- Informed energy policies: regardless of whether stock market returns are significantly affected by oil supply dynamics or other factors, policymakers can refine energy security measures to strengthen resilience against oil supply disruptions.
- Reorientation of monetary policies: central banks may consider adjustments in interest rates and policy tools to ensure economic stability during periods of turbulence in oil markets.
- Improving market supervision: financial regulators can use insights from the study to assess the need for tailored market regulations; this could include closely monitoring for market manipulation or developing mechanisms to strengthen market resilience during periods of intense volatility in the oil market.
- Investor education: investor education initiatives might equip investors with the skills needed to navigate the complexities of stock markets during periods of oil market volatility, providing knowledge on possible impacts and risk management strategies.
- International collaboration: collaborative efforts to address oil market risks can amplify the effectiveness of policies aimed at mitigating economic and financial impacts.
- Accelerating the energy transition process: the results highlight the urgency of transitioning to sustainable, less oil-dependent energy sources. Policymakers could be pushed to accelerate efforts to promote renewable energy and reduce dependence on fossil fuels.

In summary, the study’s findings carry broad policy implications spanning financial regulations, economic policies, energy strategies, and investor education. Policymakers and regulators would do well to consider these insights as they strive to strengthen economic stability and market resilience in the face of oil market fluctuations.

In conclusion, the findings of this study provide remarkable insights into the complex relationship between oil prices and European stock returns. These results have important implications for policymakers, investors, and firms who are interested in understanding the linkages between energy markets and the broader economy. After the Russian invasion of Ukraine, the prices of natural resources and energy costs sharply increased, with a turbulent phase in financial markets. In fact, the conflict generated a sharp increase in electricity prices and significant volatility in energy markets. As a consequence, the EU introduced economic sanctions targeting the Russian energy industry, most notably the coal and oil sectors. Therefore, a strong link between oil prices and stock market returns persists (Magazzino et al. 2023; Magazzino and Mele 2022).

Future research may perform alternative empirical strategies (i.e., machine learning, artificial neural networks, panel VAR, quantile regressions, and WA).

## Figures and Tables

**Fig. 3:**
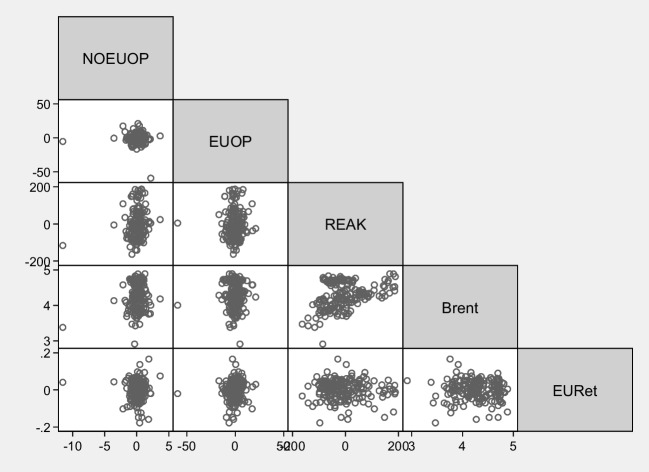
Scatterplot matrices. Sources: authors’ elaborations in STATA

## Data Availability

The data that support the findings of this study are available from the corresponding author upon reasonable request.

## Appendix

Table 7 and Fig. 3

**Table 7:** Correlation matrix

Variable	NOEUOP	EUOP	REAK	Brent	EURet
NOEUOP	1.0000				
EUOP	− 0.0507 (0.9990)	1.0000			
REAK	0.1490 (0.3790)	0.0118 (1.0000)	1.0000		
Brent	0.1660 (0.2345)	0.0190 (1.0000)	− 0.3612*** (0.0000)	1.0000	
EURet	− 0.0267 (1.0000)	0.1189 (0.6977)	− 0.0427 (0.9998)	− 0.0328 (1.0000)	1.0000

Sidak’s correction has been applied, *p*-values in parentheses. ****p* < 0.01, ***p* < 0.05, **p* < 0.10
